# Geographic and socioeconomic inequalities in the survival of children under-five in Nigeria

**DOI:** 10.1038/s41598-022-12621-7

**Published:** 2022-05-19

**Authors:** Chijioke Ifeanyi Okoli, Mohammad Hajizadeh, Mohammad Mafizur Rahman, Rasheda Khanam

**Affiliations:** 1 School of Business, and Centre for Health Research, University of Southern Queensland, Toowoomba, QLD 4350 Australia; 2 Department of Health Administration and Management, Faculty of Health Sciences and Technology, College of Medicine, University of Nigeria, Enugu Campus, Enugu, Enugu State Nigeria; 3 School of Health Administration, Dalhousie University, Halifax, Canada

**Keywords:** Health care economics, Public health

## Abstract

Despite a substantial decline in child mortality globally, the high rate of under-five mortality in Nigeria is still one of the main public health concerns. This study investigates inequalities in geographic and socioeconomic factors influencing survival time of children under-five in Nigeria. This is a retrospective cross-sectional quantitative study design that used the latest Nigeria Demographic Health Survey (2018). Kaplan–Meier survival estimates, Log-rank test statistics, and the Cox proportional hazards were used to assess the geographic and socioeconomic differences in the survival of children under-five in Nigeria. The Kaplan–Meier survival estimates show most under-five mortality occur within 12 months after birth with the poorest families most at risk of under-five mortality while the richest families are the least affected across the geographic zones and household wealth index quintiles. The Cox proportional hazard regression model results indicate that children born to fathers with no formal education (HR: 1.360; 95% CI 1.133–1.631), primary education (HR: 1.279; 95% CI 1.056–1.550) and secondary education (HR: 1.204; 95% CI 1.020–1.421) had higher risk of under-five mortality compared to children born to fathers with tertiary education. Moreover, under-five mortality was higher in children born to mothers’ age ≤ 19 at first birth (HR: 1.144; 95% CI 1.041–1.258). Of the six geopolitical zones, children born to mothers living in the North-West region of Nigeria had 63.4% (HR 1.634; 95% CI 1.238–2.156) higher risk of under-five mortality than children born to mothers in the South West region of Nigeria. There is a need to focus intervention on the critical survival time of 12 months after birth for the under-five mortality reduction. Increased formal education and target interventions in geopolitical zones especially the North West, North East and North Central are vital towards achieving reduction of under-five mortality in Nigeria.

## Introduction

Globally, geographic and socioeconomic differences in childhood mortality are a major public health concern, especially in low- and middle-income countries (LMICs)^1–3^. Geographic inequalities in mortality in LMICs are usually substantial and partly explain socioeconomic differences in childhood mortality^4^. According to the World Health Organization (WHO), children in developing countries are 10 times more likely to die before their fifth birthday compared with children in developed countries^2,5^. In sub-Saharan Africa regions, 1 in 8 children dies before age five, which is nearly 20 times the average of 1 in 167 for developed regions^5,6^.

There has been a substantial decline in childhood mortality on the global stage^7^. The high rate of childhood mortality in Nigeria (132 deaths per 1000 births) is still one of the main public health concerns^8^. In Nigeria, every minute one child under-5 years dies i.e. 1440 deaths daily^9^. Given that the childhood mortality rate is a major indicator of the overall health of a population and level of economic development of any country^10,11^, Nigeria requires a minimum target of 44% reduction in under-five mortality rates (U5MR) every 5 years to reach the target of < 25 U5MR of the Sustainable Development Goal (SDG) by the year 2030^12^. This involves a concerted and sustainable effort in removing various barriers to healthcare access and utilization^13^. However, the public health concern is the lack of attention to the large and growing inequities in the provision of and access to services within low-middle-income countries^14^.

The issue of child mortality does not draw media attention because it is occurring amongst the poorest, most vulnerable, and voiceless communities^12^. Differential distribution of child mortality also exists across different geographical regions in LMICs. In Nigeria, for example, U5MR varies across the six geopolitical zones (North Central, North East, North West, South East, South South, and South West), ranging from 89 deaths per 1000 births in the South West to 222 deaths per 1000 births in the North East^6^. The country loses far too many under-five children, making it one of the largest contributors to global under-five mortality rates^15^.

To date, some cross-sectional studies have analysed geographic and socioeconomic determinants of U5MR in Nigeria^6,10,16,17^. However, these studies did not take into consideration the time-to-event censured data analysis. To address this gap in the literature, this study uses the Kaplan–Meier survival estimates and the Cox proportional-hazards regression model instead of the traditional regression model to assess socioeconomic and geographical differences in U5MR. The Kaplan–Meier and the Cox proportional-hazards regression models are robust and preferred over the logistic regression, which ignores survival time and censoring information^18–21^. Since the major causes of child mortality are largely avoidable and/or preventable^22^, identifying the geographic and socioeconomic factors influencing U5MR in Nigeria helps policy and decision-makers to understand differences in U5MR between the most and least deprived social and geographic groups who may consequently benefit from focused interventions to prevent child mortality.

## Methods

### Study setting

The study setting is Nigeria, with an estimated population of around 200 million people and by implication the most populous sub-Saharan African country^9^. Administratively, the country is divided into six geopolitical zones, comprising 36 states and the Federal Capital Territory, Abuja. The country is culturally, ethnically, and geographically heterogeneous, with more than 250 identifiable ethnic groups^16^. Of the ethnic groups, the largest and politically dominant ethnic groups are the Hausa (North), the Igbo (South East), and the Yoruba (South West)^23^. Cultural values and practices unique to ethnic groups influence child health outcomes. For example, Yoruba and Igbo girls tend to marry in the third decade of life, while early marriage, before age 16 years, is common among the Hausa/Fulani/Kanuri ethnic groups^23^. The proportion of educated people is high among Igbo, Yoruba, and minority ethnic groups compared to the less-educated Hausa/Fulani/Kanuri tribes^16^. While more than 70% of the northern geopolitical zones live below the poverty line, this figure is less than 35% in the southern geopolitical zones^24^. Due to the cultural differences among Nigerians, there are significant ethnic variations in health care utilization in the country^25^. Nevertheless, many primary health facilities have fallen into disrepair and most public services are not trusted due to poor service delivery^9^.

### Study design

This is a retrospective cross-sectional quantitative study using the latest 2018 Nigeria Demographic and Health Survey (2018 NDHS) conducted from August 14, 2018 to December 29, 2018^8^.

### Data source

The NDHS is a nationally representative health survey conducted every 5 years using a multistage sampling procedure, standardized tools and well-trained interviewers^8^. It is the world's largest survey, eliciting information on infant and child mortality rates and demographic factors generated from birth histories obtained from the mothers interviewed^2^. The DHS also contains information on socioeconomic and geographic characteristics including household ownership of assets, maternal education, and rural/urban residence^4,26^. As measuring inequalities in childhood mortality requires information on age at death of under-five, and socioeconomic status, the 2018 NDHS Birth Recode data file was used. It contained 127,545 sample sizes with a response rate of 99%^8^. Analysis in this study was restricted to children aged 0–59 months (n = 33,741).

### Variables

The dependent variable is descriptive binary (1 if a live-born child dying before its fifth birthday, 0 otherwise) plus time in months until the event (death) occurs. In line with previous studies^19,21,25,27,28^, the independent variables comprise child gender, mother’s age (at first birth), household wealth index, maternal and paternal education (as an indicator for socioeconomic status), ethnic origin, religion, place of residence, and geopolitical zone. Table 1 presents the description of variables used in the analysis.

**Table 1:** Description of variables used in the study.

Dependent variable	Variable description
Under-five mortality (event)	1 = if the child died, 0 otherwise
**Age of child (in months)**	
< 1 month (neonatal)	The child died before one month
1–11 months (post-neonatal)	The child died between 1–11 months
12–59 months (childhood)	The child died between 12 to 59 months
**Independent variables**	
Demographic variables	
Child’s gender	
Male	1 = if the child is a male, 0 if a child is a female
Mother’s age (at first birth)	
≤ 19	1 = if the mother’s age at first birth is ≤ 19, 0 otherwise
20–39	1 = if the mother’s age at first birth is between 20 and 39, 0 otherwise
≥ 40	1 = if the mother’s age at first birth is ≥ 40, 0 otherwise
Ethnic origin	
Hausa/Fulani/Kanuri	1 = if the maternal ethnic origin is Hausa/Fulani/Kanuri, 0 otherwise
Igbo	1 = if the maternal ethnic origin is Igbo, 0 otherwise
Yoruba	1 = if the maternal ethnic origin is Yoruba, 0 otherwise
Others	1 = if the maternal ethnic origin is not Hausa, Igbo or Yoruba, 0 otherwise
Religion	
Christian	1 = if the mother is a Christian, 0 otherwise
Muslim	1 = if the mother is a Muslim and others, 0 otherwise
**Socioeconomic variables**	
Maternal education	
No formal education	1 = if the mother has no formal education, 0 otherwise
Primary education	1 = if the mother has a primary education, 0 otherwise
Secondary education	1 = if the mother has a secondary education, 0 otherwise
Higher education	1 = if the mother has higher education, 0 otherwise
Paternal education	
No formal education	1 = if the father has no formal education, 0 otherwise
Primary education	1 = if the father has a primary education, 0 otherwise
Secondary education	1 = if the father has a secondary education, 0 otherwise
Higher education	1 = if the father has higher education, 0 otherwise
Wealth index (household)	
Poorest (1)	1 = if the mother is in poorest quintile, 0 otherwise
Poorer (2)	1 = if the mother is in poorer quintile, 0 otherwise
Middle (3)	1 = if the mother is in middle quintile, 0 otherwise
Richer (4)	1 = if the mother is in richer quintile, 0 otherwise
Richest (5)	1 = if the mother is in richest quintile, 0 otherwise
**Geographic and geopolitical variables**	
Place of residence	
Urban residence	1 = if the mother lives in an urban area, 0 otherwise
Geopolitical zone	
North Central	1 = if the mother is from North Central, 0 otherwise
North East	1 = if the mother is from North East, 0 otherwise
North West	1 = if the mother is from North West, 0 otherwise
South East	1 = if the mother is from South East, 0 otherwise
South South	1 = if the mother is from South South, 0 otherwise
South West	1 = if the mother is from South West, 0 otherwise

### Statistical analysis

We used a survival analysis method to examine survival time-to-event data. Of the three survival analysis techniques (parametric modelling, semiparametric modelling and nonparametric analysis), we adopted the later (nonparametric) for analysing censored data^21^ and for it capacity of letting the dataset speak for itself^29^. We used the two most common nonparametric methods in survival analysis viz., Kaplan–Meier survival estimates and Log-rank test statistic to assess the survival function and pattern of under-five mortality^20,30–32^.

The Kaplan–Meier survival estimates were used to present graphically a survival curve that plots survival probability against time^20^. The conditional probability of a child’s survival increases as he/she progresses in age^33^. Kaplan–Meier provides a useful summary of the data that can be used to estimate measures such as median survival time^34^.

Survival data are modelled in terms of two related functions:-, the survivor function and the hazard function^34^. Assume $T$ to be a random variable representing the survival time of subjects in the population, and $t$ be the realization of $T$. The cumulative distribution function of $T$ is expressed as:

1 $$F\left(t\right)=P\left(T<t\right).$$

where $t$ denotes the actual survival time of a child, $T$ indicates a random variable associated with the survival time, and $F(t)$ is the probability density function for the survival time.

The distribution function of $T$ and survival function $S\left(t\right)$ show the proportion of children that survive longer than $t$ from the first day of birth and is expressed as:

2 $$F\left(t\right)=P\left(T>t\right)= \int\limits_{0}^{t}f\left(u\right)du$$

3 $$S\left(t\right)=P\left(T \ge t)=1-F(t\right)$$

The hazard function, $h\left(t\right)$, represents the probability that an individual dies at a time, conditional on having survived to that time. That is, the function represents the instantaneous death rate for an individual surviving to time $t$:

4 $$h\left(t\right)=\underset{\Delta \to 0}{\mathrm{lim}}\frac{P(t<T\le t+\Delta t\backslash T>t}{\Delta t}=\frac{f(t)}{S(t)}.$$

where $h\left(t\right)$ is the hazard function, $T$ is the survival time,$S\left(t\right)$ is the survival function, and $\Delta$ is the instantaneous change^19,21^.

The Cox proportional hazards regression model, the most widely used model in the analysis of survival data, was used to assess the influence of various covariates in the survival times of individuals through the hazard function^19^. It provides a hazard ratio to compare survival times of two or more population groups. The observation is right-censored, that is the survival status of the individual might not be known at the time of the survey^19,25^. In the model, the exponentiated linear regression portion of the model explains the effects of explanatory variables on hazard ratio^19^.

The Cox hazard is modelled as follows:

5 $$h\left(t\right)={h}_{0 }\left(t\right)exp.\left({b}_{1}{X}_{1}+{b}_{2}{X}_{2}+{b}_{3}{X}_{3}+\cdots {b}_{k}{X}_{k}\right),$$

where ${X}_{1}$ to ${X}_{k}$ are $k$ explanatory variables and ${h}_{0}(t)$ is the baseline hazard at time $t$, representing the hazard for a person with the value 0 for all the explanatory variables. By dividing both sides of Eq. (5) by ${h}_{0}(t)$ and taking logarithms, we obtain:

6 $$ln\frac{h(t)}{{h}_{0}(t)}={b}_{1}{X}_{1}+{b}_{2}{X}_{2}+{b}_{3}{X}_{3}+\cdots +{b}_{k}{X}_{k},$$

where $\frac{h(t)}{{h}_{0}(t)}$ is the hazard ratio. The coefficients ${b}_{1}\;\text{to}\;{b}_{k}$ are estimated by the Cox regression^25^.

Three models were fitted into the Cox proportional hazards model for analysis to investigate the influence of the predictor variables on the under-five mortality. Model 1 contained demographic factors as the only predictor variables while model 2 added socioeconomic variables. Model 3 included environmental and geopolitical variables along with the previous variables in models 1 and 2 for analysis^21^.

In the Cox proportional hazards model, the outcome is described in terms of the hazard ratio. The Cox regression model gives the hazard function as a product of a baseline hazard involving $t$ and an exponential expression involving $X{^{\prime}}s$ without $t$. The exponential part of the equation ensures that the fitted model will always give estimated hazards that are non-negative^35^.

The hazard ratio represents the instantaneous risk over the study time. The measures of association were expressed as hazard ratios (HR) with 95% confidence intervals (CI)^20^. A hazard ratio of 1 means lack of association, a hazard ratio > 1 suggests an increased risk and a hazard ratio < 1 suggests a smaller risk. In general, the survivor function focuses on not having an event, while the hazard function focuses on the event occurring^34^. All analyses were weighted to ensure representativeness of the survey sample. A p-value less than 0.05 is considered statistically significant. We conducted all the analysis using Stata version 13.1. More so, all methods were performed in accordance with the relevant guidelines and regulations.

### Ethical approval and consent to participate

The Demographic and Health Surveys (DHS) Program has granted approval to use the Nigeria DHS dataset for this study. The DHS adhered to informed consent and we observed anonymity and confidentiality under the data terms of use.

## Results

Table 2 presents the descriptive statistics of variables used in the study and distribution of under-five (0–59 months) deaths across the demographic, geographic and socioeconomic characteristics. The results show that the proportion of under-five mortality (U5M) is about 10%, and higher among the male (51.0%) than the female (49.0%) gender. Although the proportion of age of child (in months) is highest (82.2%) among the childhood (i.e. 12–59 months) group, the proportion from total U5M is the highest (39.5%) among the neonatal (< 1 month) age bracket.

**Table 2:** Descriptive statistics of variables used in the study and distribution of under-five (0–59 months) deaths across the demographic, geographic and socioeconomic characteristics.

Variable	Proportion	Proportion from total under-five deaths
Under-five mortality (event)	9.7%	
**Age of child (in months)**		
< 1 month (neonatal)	0.9%	39.5%
1–11 months (post-neonatal)	16.9%	27.7%
12–59 months (childhood)	82.2%	32.8%
**Demographic variables**		
Child's gender		
Male	51.0%	53.5%
Female	49.0%	46.5%
Mother’s age (at first birth)		
≤ 19	58.0%	67.2%
20–39	41.9%	32.7%
≥ 40	0.1%	0.1%
Ethnic origin		
Hausa/Fulani/Kanuri	48.9%	57.9%
Igbo	12.6%	9.2%
Yoruba	11.0%	5.8%
Others	27.5%	27.1%
Religion		
Christian	35.9%	28.0%
Muslim	64.1%	72.0%
**Socioeconomic variables**		
Maternal education		
No formal education	46.5%	58.1%
Primary	14.9%	15.4%
Secondary	30.4%	22.2%
Tertiary	8.2%	4.3%
Paternal education		
No formal education	37.5%	49.1%
Primary	14.1%	14.9%
Secondary	33.7%	27.5%
Tertiary	14.7%	8.5%
Wealth index (household)		
Poorest (1)	22.2%	30.7%
Poorer (2)	22.6%	28.0%
Middle (3)	20.6%	20.5%
Richer (4)	18.4%	13.2%
Richest (5)	16.2%	7.6%
**Environmental variables**		
Place of residence		
Urban	38.6%	26.5%
Rural	61.4%	73.5%
Geopolitical zones		
North Central	13.5%	14.8%
North East	18.3%	22.8%
North West	36.5%	42.4%
South East	10.1%	7.9%
South South	8.7%	5.7%
South West	12.9%	6.4%
Sample size	33,741	3194

Further, the result indicates that the proportion of U5M is prevalent among children born to mothers’ age ≤ 19 at first birth (58.0%), especially among the Hausa/Fulani/Kanuri ethnic origin (48.9%) and the Muslim groups (64.1%). Moreover, the socioeconomic variables show that the U5M was higher among children born to mother (46.5%) and father (37.5%)] with no formal education compared to the children born to mother or father with primary, secondary, or tertiary education. In the same vein, of the household wealth index quintiles, 65.4% of the under-five death occurred among the three lowest wealth quintiles: poorest (22.2%), poorer (22.6%) and middle (20.6%).

About 61.4% of the U5M occurred in rural areas, while the remaining 38.6% occurred in urban areas in Nigeria. Of the six geopolitical zones, 68.3% of U5M occur in the three northern geopolitical zones: North West (36.5%), North East (18.3%), and North Central (13.5%)] compared to their southern counterparts (31.7%). The majority of U5M occurs among those residing in rural areas (73.5%).

**Figure 1:**
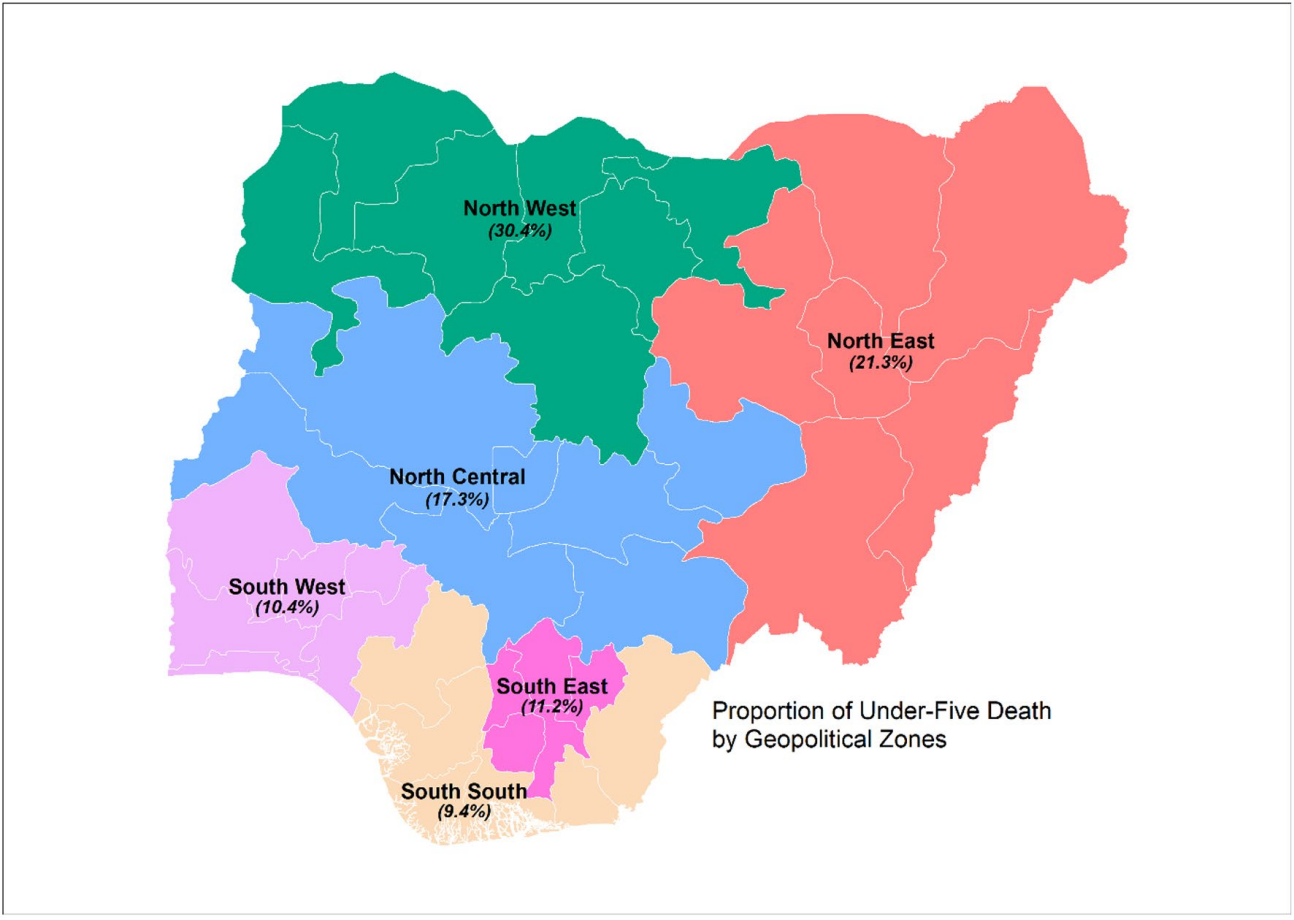
Proportion of under-five (U5) death by geopolitical zones of Nigeria (Source: http://www.gadm.org (data); software: ArcMap version 10.8.1 (Esri), http://www.esri.com).

Figure 1 presents proportion of under-five death by geopolitical zones in a Nigeria map. Of the six geopolitical zones, North West (30.4%) followed by the North East (21.3%) and North Central (17.3%), had the highest under-five death compared to the South East (11.2%), South West (10.4%) and South South (9.4%).

**Figure 2:**
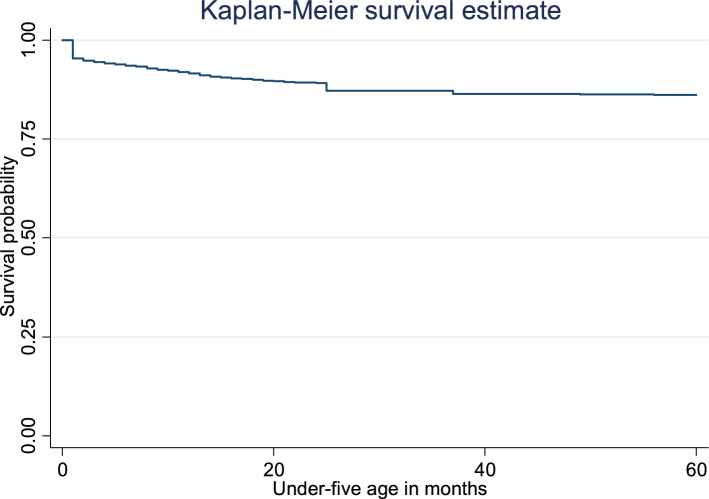
Plot of the overall estimate of the Kaplan–Meier survivor function of under-five mortality in Nigeria.

Figure 2 shows the Kaplan–Meier estimates of the survival graph for all the under-five children. The horizontal axis indicates the time to event in months, while the vertical axis shows the survival probability or the proportion of under-five children surviving. At time 0, the survival probability is 1.00 (i.e. 100% of the participants are alive). Thus, the result indicates most under-five death occurs at earlier months after birth.

**Figure 3:**
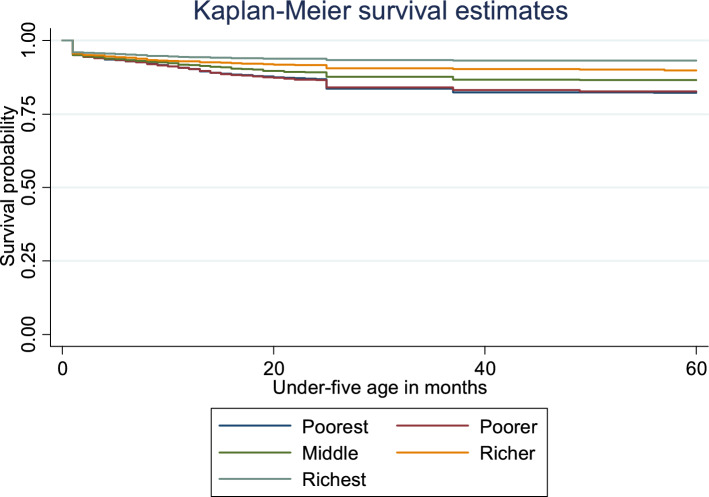
Plot of the Kaplan–Meier survival estimates of under-five mortality by household wealth index quintiles in Nigeria, 2018.

Figure 3 graphically presents Kaplan–Meier survival estimates of under-five mortality by household wealth index quintiles. The graph shows that the top wealth quintiles had higher under-five survival probability than the bottom quintiles. The survival probability is high for the richest but relatively low for the poorest. Even so, the survival probability of the poorer and the poorest were almost the same. The statistically significant value of the log-rank test for equality of survivor functions for household wealth index (χ^2^ = 217.89 P < 0.001) indicates differences in survival probability among different socioeconomic groups.

**Figure 4:**
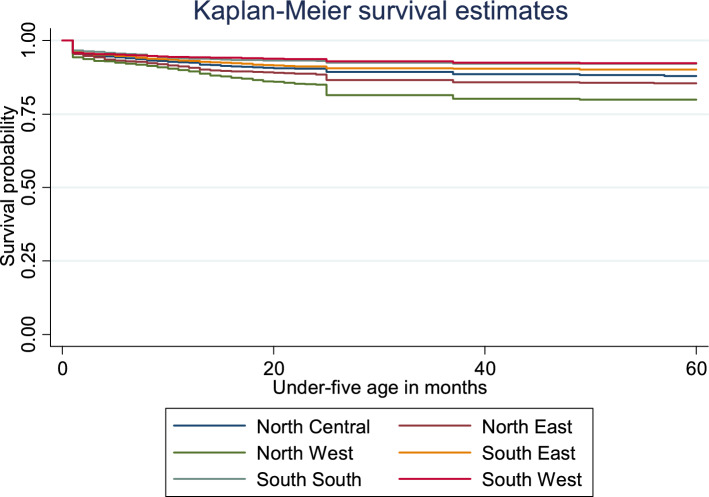
Plot of the Kaplan–Meier survival estimates of under-five mortality by geopolitical zones in Nigeria, 2018.

Figure 4 plots the Kaplan–Meier survival estimates of under-five mortality by geopolitical zones. The graph indicates that the North West followed by the North East and the North Central have the most at risk of survival among the six geopolitical zones while South South, South East and South West have a lower risk of survival. The geographic zones log-rank test for equality of survivor functions is statistically significant (χ^2^ = 307.45, P < 0.001).

Table 3 presents the results of the Cox proportional hazard regression analysis. It assesses the influence of different factors on the survival time of under-five children. The demographic variables included in Model 1 assessed the independent influence of demographic factors on the risk of under-five deaths. The result shows that U5M is 15.5% higher among male children (HR: 1.155; 95% CI 1.070–1.247, p < 0.001) than female children. Moreover, U5M is 25.4% higher in children born to mothers age ≤ 19 at first birth (HR: 1.254; 95% CI 1.148–1.369, p < 0.000) than children born to mothers age 20–39 at first birth. Further, the results show that mortality by ethnic origin is 47.8% (HR: 0.522; 95% CI 0.437–0.625, p < 0.001); 24.7% (HR: 0.753; 95% CI 0.625–0.907, p < 0.003) and 25.9% (HR: 0.741; 95% CI 0.661–0.830, p < 0.000) lower for children whose mothers are from the Yoruba, Igbo and other ethnic origin respectively, compared to the children whose mothers are of Hausa/Fulani/Kanuri origin. More so the U5M is 26.2% higher among children born to Muslim mothers (HR: 1.26.2; 95% CI 1.110–1.434, p < 0.001) than children born to Christian mothers.

**Table 3:** The Cox's proportional hazard ratios (HR) with 95% confidence interval (CI) for factors affecting under-five mortality in Nigeria 2018.

Variables	Model 1	Model 2	Model 3
	HR (95% CI)	HR (95% CI)	HR (95% CI)
**Demographic factors**			
Child's gender			
Female (ref)	1	1	1
Male	1.155 (1.070–1.247)**	1.152 (1.064–1.247)**	1.154 (1.066–1.250)**
Mother’s age (at first birth)			
≤ 19	1.254 (1.148–1.369)**	1.157 (1.052–1.271)**	1.144 (1.041–1.258)**
20–39 (ref)	1	1	1
≥ 40	1.604 (0.400–6.427)	1.785 (0.445–7.161)	1.836 (0.457–7.371)
Ethnic origin			
Hausa/Fulani/Kanuri (ref)	1	1	1
Igbo	0.753 (0.625–0.907)**	0.877 (0.716–1.075)	1.122 (0.789–1.596)
Yoruba	0.522 (0.437–0.625)**	0.667 (0.548–0.812)**	0.908 (0.684–1.205)
Others	0.741 (0.661–0.830)**	0.797 (0.708–0.896)**	0.957 (0.834–1.099)
Religion			
Christian	1	1	1
Muslim	1.262 (1.110–1.434)**	1.220 (1.061–1.403)**	1.147 (0.990–1.328)*
**Socioeconomic factors**			
Maternal education			
No formal education		0.971 (0.751–1.256)	0.962 (0.744–1.244)
Primary		1.000 (0.777–1.288)	1.011 (0.785–1.301)
Secondary		0.941 (0.750–1.182)	0.953 (0.759–1.196)
Tertiary (ref)		1	1
Paternal education			
No formal education		1.328 (1.107–1.592)**	1.360 (1.133–1.631)**
Primary		1.252 (1.035–1.515)**	1.279 (1.056–1.550)**
Secondary		1.180 (1.000–1.392)**	1.204 (1.020–1.421)**
Tertiary (ref)			
Wealth index (household)			
Poorest (1) (ref)		1	1
Poorer (2)		1.080 (0.971–1.201)	1.064 (0.956–1.184)
Middle (3)		0.956 (0.843–1.083)	0.987 (0.868–1.122)
Richer (4)		0.804 (0.689–0.937)**	0.865 (0.735–1.018)**
Richest (5)		0.639 (0.522–0.783)**	0.703 (0.567–0.872)**
**Environmental factors**			
Place of residence			
Urban (ref)			1
Rural			1.087 (0.974–1.212)
Geopolitical zones			
North Central			1.199 (0.927–1.552)
North East			1.221 (0.928–1.608)
North West			1.634 (1.238–2.156)**
South East			1.072 (0.726–1.581)
South South			0.946 (0.701–1.277)
South West (ref)			1
Log rank test (p-value)			χ^2^ = 307.45 (p < 0.001)

**p < 0.05, *p < 0.1.

Model 2 examines the influence of demographic and socioeconomic variables on the risk of under-five deaths. Results indicate that U5M is 15.2% higher for male children (HR: 1.152; 95% CI 1.064–1.247, p < 0.001) than females. In addition, U5M is 15.7% higher in children born to mothers aged ≤ 19 at first birth (HR: 1.157; 95% CI 1.052–1.271, p < 0.003). Children of Yoruba (HR: 0.667; 95% CI 0.548–0.812, p < 0.000) and other ethnic origins (HR: 0.797; 95% CI 0.708–0.896, p < 0.001) had 33.3% and 20.3%, respectively lower U5M compared with the Hausa/Fulani/Kanuri ethnic origin. About 22.0% U5M occurs among children of Muslim mothers than children of Christian mothers. These findings show a significant association between U5M and paternal education. Results show that children born to fathers with no formal education (HR: 1.328; 95% CI 1.107–1.592, p < 0.002), primary education (HR: 1.252; 95% CI 1.035–1.515, p < 0.021) and secondary education (HR: 1.180; 95% CI 1.000–1.392, p < 0.050) had 32.8%, 25.2% and 18.0%, respectively, have a higher risk of U5M compared to children born to fathers with tertiary education. Of the household wealth index quintile, the richest (HR: 0.639; 95% CI 0.522–0.783, p < 0.001) and richer (HR: 0.804; 95% CI 0.689–0.937, p < 0.005) groups had 36.1% and 19.6%, respectively, lower risk of U5M compared with the poorest quintile group.

Finally, the results of Model 3 for demographic factors were consistent with Models 1 and 2 results. In the same vein, results indicate that children born to fathers with no formal education (HR: 1.360; 95% CI 1.133–1.631, p < 0.001), primary education (HR: 1.279; 95% CI 1.056–1.550, p < 0.012) and secondary education (HR: 1.204; 95% CI 1.020–1.421, p < 0.028) had 36.0%, 27.9% and 20.4%, respectively, higher risk of U5M compared to children born to fathers with tertiary education. Moreover, results suggest that the richest (HR: 0.703; 95% CI 0.567–0.872, p < 0.001) and richer (HR: 0.865; 95% CI 0.735–1.018, p < 0.082) wealth quintile groups had 29.7% and 13.5%, respectively, lower risk of U5M compared with the poorest quintile. Of all the geopolitical zones, children born to mothers living in the North West (HR 1.634; 95% CI 1.238–2.156, p < 0.001) had 63.4% higher risk of U5M than South West zone.

## Discussion

Evidently, under-five mortality rate (U5MR) is the highest in sub-Saharan African countries and Nigeria in particular. Notwithstanding that, these deaths are preventable in part by addressing the associated demographic, geographic, and socioeconomic factors^18^. This study investigates the geographic and socioeconomic survival differences of under-five in Nigeria.

Findings from the Kaplan–Meier survival estimates show the most U5Ms occur within 12 months after birth with the poorest most at risk of U5M while the richest are the least affected across household wealth index quintiles. The findings are in tandem with the UNICEF report that under-five deaths are increasingly concentrated in the neonatal period^5,36^. Besides, our finding is in line with the assertion by Lartey and colleagues that the probability of a child’s survival increases as the child progresses in age and that the survival probability is lower for children from the poorest families but higher for the children from the richest families^33^. Therefore, U5M reduction interventions may target children under 12 months of birth given their fragile immune systems. This is to protect them from the perennial environmental threats to child health such as malaria (from mosquito bites), lack of clean water, and poor sanitation^2,17^.

Further, the finding shows that of the six geopolitical zones in Nigeria, the northern zones especially North West, North East and North Central are at the most risk of U5M. Earlier studies^6,23,37^ corroborate that the risk of under-five deaths is higher in the North West and North East regions owing to higher proportions of home delivery and complications during childbirth, younger age at birth of first child, and poor utilization of modern health facilities compared to the southern region. The finding establishes that geopolitical setting strongly influence the health and survival chances of children^1^, thus, implying that U5M risks could be contained depending on the geopolitical environment children in which find themselves^16^.

As a corollary, our study shows that children born to mothers living in rural areas experience higher U5M compared to their urban counterparts. Often children born to poor mothers in rural areas are delivered at home^38^. This is not surprising as modern health care is not easily available in rural areas as in the urban area, hence, urban areas are reported to have lower U5Ms than rural areas^6,18,27^.

The Cox proportional hazard regression models show that paternal education is negatively related to U5M: increased paternal education leads to a reduction in U5M and vice versa. This is in tandem with the assertion that parental education increases a child’s survival probability^30^. However, contrary to expectations the maternal education was not statistically significant although mothers’ education has a relatively higher impact on child mortality than fathers’ education and many other socioeconomic factors^27,33,38,39^. This could be due to the finding that mothers in northern Nigeria have a higher proportion of no education or primary education^37^ due to early marriage (before age 16 years)^23^ and combined with the fact that culturally, husbands are the overall decision-makers and breadwinner, especially in the regions^40^.

Moreover, the Cox regression model of the household wealth index shows that the rich had lower risk of U5M compared to the poor as shown in Cox models 2 and 3. A study in India affirms that the risk of child mortality is the highest among the poor^41^. It presupposes that a targeted intervention to the poor is necessary to close the gap.

This study also shows that children from Hausa/Fulani/Kanuri ethnic origin (northern region) experience more under-five mortality compared to the southern region. Earlier studies in Nigeria, indicates that a child born in the North West has a 2.5 times higher probability of dying before age five than one born in the South East^42^. This could be due to the preponderance of early marriage commonly practiced in northern Nigeria. Thus, given that education is a fundamental factor to consider in terms of child survival irrespective of region, formal education sensitization particularly in northern Nigeria would help alleviate childhood mortality in the country^6^.

The results consistently indicate that male children have higher mortality compared with female children. This is supported by literature that males have a higher mortality in infancy in both Nigeria and globally^43^. It brings to fore the findings of Wegbom and colleagues that U5M in Nigeria is diversely affected by health-related factors and non-health sector factors such as demographic, economic, environmental, social, and security^21^. Therefore, it requires a health equity-in-all policies approach to tackle under-five mortality^44^.

The strength of this work is the use of the Kaplan–Meier survival estimates and Cox proportional hazard model for analysing time-to-event data and censored observations. Nevertheless, this study is subject to some limitations. Firstly, although DHS is a renowned reliable data source on child mortality^2,36^, we acknowledge the inherent data collection challenges that could manifest through misreporting of age of child or age at death in months. Secondly, our analyses focused on U5M over the last 5 years, and the household wealth index constructed for the survey year was used as one of the proxies for socioeconomic status. Since, changes in household wealth often occur in the long-run, the current measures of wealth index can be a valid proxy for past values^45^. Notwithstanding this, the current measure of both dependent and independent variables would have been ideal for the analysis. Lastly, as our analyses were based on retrospective cross-sectional data, temporality could not be established between explanatory variables and geographic or socioeconomic inequality in U5M; thus, impeding causal inference.

## Conclusion

Achieving a reduction in U5M is a public health concern that requires a multi-dimensional approach. There is a need to tailor U5M reduction interventions to the critical survival time of 12 months after birth. A target intervention in geopolitical zones especially the North West, North East and North Central will be of utmost importance to increase access to needed health care services. In addition, increased formal education particularly in northern Nigeria is vital for U5M reduction in the country, given that education is a fundamental factor to consider in terms of child survival irrespective of region.

## Data Availability

Data for this study is publicly accessible upon request from the DHS website: https://www.dhsprogram.com/data/available-datasets.cfm.

## Abbreviations

CI: Confidence interval
DHS: Demographic and Health Survey
HR: Hazard ratio
LMIC: Low-and-middle-income countries
SDGs: Sustainable development goals
U5MR: Under-five mortality rate
WHO: World Health Organization
